# Is correction for gradient nonlinearity necessary in a brain diffusion tensor MRI clinical study?

**DOI:** 10.1371/journal.pone.0350808

**Published:** 2026-07-06

**Authors:** Praitayini Kanakaraj, Tianyuan Yao, Zhiyuan Li, Nancy R. Newlin, Michael E. Kim, Chenyu Gao, Tian Yu, Aravind Krishnan, Baxter P. Rogers, Tim Hohman, Angela L. Jefferson, Niranjana Shashikumar, Kimberly R. Pechman, L. Taylor Davis, Daniel Moyer, Kurt G. Schilling, Derek Archer, Adam Anderson, Bennett A. Landman

**Affiliations:** 1 Department of Computer Science, Vanderbilt University, Nashville, Tennessee, United States of America; 2 Department of Electrical and Computer Engineering, Vanderbilt University, Nashville, Tennessee, United States of America; 3 Vanderbilt University Institute for Imaging Science, Vanderbilt University Medical Center, Nashville, Tennessee, United States of America; 4 Vanderbilt Memory and Alzheimer's Center, Vanderbilt University Medical Center, Nashville, Tennessee, United States of America; 5 Department of Neurology, Vanderbilt University Medical Center, Nashville, Tennessee, United States of America; 6 Vanderbilt Genetics Institute, Vanderbilt University School of Medicine, Nashville, Tennessee, United States of America; 7 Department of Medicine, Vanderbilt University Medical Center, Nashville, Tennessee, United States of America; 8 Department of Psychology, Vanderbilt University, Nashville, Tennessee, United States of America; 9 Department of Biomedical Engineering, Vanderbilt University, Nashville, Tennessee, United States of America; 10 Department of Radiology and Radiological Services, Vanderbilt University Medical Center, Vanderbilt University Medical, Nashville, Tennessee, United States of America; Medical Center - University of Freiburg, GERMANY

## Abstract

Nonlinear gradients alter the diffusion encoding in brain diffusion tensor imaging (DTI), leading to spatially varying diffusion weighting which bias quantitative measures if uncorrected. Although the overall effects of gradient nonlinearity correction in brain studies are typically minimal and often fall below the detection limits of traditional imaging resolutions and sensitivities, their cumulative impact on clinical outcomes requires further study. This study investigates the significance and effects of correcting gradient nonlinearity in DW-MRI, focusing on the microstructural and macrostructural changes in white matter (WM) and gray matter (GM) across a clinical cohort. Our primary aim is to clarify whether the observed nonlinearity significantly alters the interpretation of aging in clinical settings, particularly in studies comparing healthy individuals to those with neurological conditions. We assess the extent of nonlinear fields impact on individual scans, interscanner observations, and a tract-based analysis. Using data from the Vanderbilt Memory & Aging Project (n = 948 imaging sessions, 933 on Scanner B and 15 on Scanner A acquired with single-shell diffusion tensor imaging protocol), we find 1%, 3.3%, and 5-degree changes in microstructure measures, fractional anisotropy (FA), mean diffusivity (MD), and primary eigen vector (V1) respectively, affecting at least 20% of the brain. Across sessions, head positioning sampled typical clinical variability, with head offsets of approximately 0–10 mm and rotations of 0–10° relative to magnet isocenter. Subcortical regions in the superior regions, occipital lobules, and parietal lobules exhibit relatively higher impacts. Macrostructural measures show changes up to 12% after nonlinear field correction. GNL effects are 5% and 0.33% of FA and MD changes between mild cognitive impairment and controls. A simple power analysis indicates that these subtle effects of gradient nonlinearity correction can become statistically detectable in larger multi-site studies exceeding ~1000 subjects, suggesting that GNL should be considered and, where possible, corrected or at least quantified in such settings.

## 1. Introduction

Diffusion MRI (DW-MRI) is a non-invasive modality to probe *in vivo* tissue micro-structure and macro-structure by capturing the random Brownian motion of water molecules [1]. Recently, diffusion MRI has been explored as a biomarker for studying neurological conditions and the aging process [2–4]. However, it is susceptible to hardware limitations that can compromise data integrity [5,6]. One significant challenge posed by scanner hardware is gradient nonlinearity (GNL), which can cause spatial distortions in the acquired images [7]. The magnitude and spatial pattern of GNL are determined by the gradient system and depend strongly on the anatomical location relative to isocenter, with larger deviations expected at greater offsets and in body regions further from the magnet center. These distortions, if not properly addressed, can lead to inaccuracies in the analysis of both microstructural and macrostructural changes within brain tissues [8–10], potentially skewing clinical assessments. In contrast to susceptibility- and eddy-current–induced geometric distortions, which primarily displace voxels in the reconstructed images, GNL in diffusion MRI mainly affects the diffusion weighting by causing spatial variations in the effective b-values and diffusion-encoding directions. In this work we focus on correcting these diffusion-encoding errors, which in turn reduce spatial biases and intensity inhomogeneities in the diffusion images.

Previous studies have identified the impact of GNL on diffusion sensitization measurements and have underscored the necessity for correction methodologies. Tan et al. observed reductions in ADC errors for a temperature-controlled isotropic diffusion phantom, with decreases from 5.8% to 0.15% at isocenter and from 10% to 0.63% at 11 cm from the isocenter [11]. Similarly, Malyarenko et al. demonstrated with a regular grid phantom that using GNL correction significantly reduces the apparent diffusion coefficient (ADC) bias across various orientations, decreasing it from approximately 20% to 2% in clinically relevant offsets for both isotropic and anisotropic media [12]. In a separate study by the same group, noted GNL corrections substantially improved interplatform accuracy from 6% to 0.5% and reproducibility from 11% to 2.5% [13]. These phantom-based studies emphasize that gradient nonlinearity can introduce large, spatially varying errors in ADC if left uncorrected. Further emphasizing GNL correction benefits in body diffusion MRI, Newitt et al. and Malyarenko et al. reported substantial spatial variation in breast ADC in multi-site breast DWI studies, with site- and position-dependent errors ranging from approximately 1.4% to 19.9% and showing strong dependence on right–left and anterior–posterior offsets from isocenter; applying scanner-specific GNL corrections reduced mean ADC errors from ~10% to well below 1% [12,14]. Tao et al. found that post-GNL correction, ADC values were typically within 2.5% of reference values across the entire field of view for various polyvinylpyrrolidone concentrated phantom [15]. Complementing these body and phantom studies, Mulkern et al. performed a multi-center isotropic phantom study demonstrating that site-to-site variability in ADC measurements is limited by gradient nonlinearity and that scanner-specific calibration and correction can substantially improve ADC standardization across clinical scanners [16]. Rogers et al. indicated that at 80 mm from the isocenter, the predicted b-value errors due to gradient nonlinearity ranged from 1% to 6%, with gradient directions potentially off by up to 1 degree [17]. The spatial variation in estimated mean diffusivity of an isotropic phantom improved from 2.2%−4.1% within 60 mm of the isocenter to 0.5%−1.6% post-correction [18]. The group-level relevance of these distortions has been particularly noted in comparisons between male and female subjects. Mesri et al.’s research emphasizes the potential impact of GNL on observed gender differences by analyzing regional diffusion parameters for both genders in brain MRI. The findings reveal that failing to correct for GNL could alter the significance and effect sizes in group-wise t-tests across various regions of interest. This suggests that some previously observed gender differences might be artifacts of uncorrected GNL rather than true anatomical variations [9]. These studies collectively suggest that uncorrected gradient nonlinearity can significantly distort DW-MRI data, especially towards the periphery of the field of view, highlighting the critical importance of applying GNL corrections to enhance accuracy and reproducibility in multicenter clinical trials across multiple scanner systems. Collectively, these studies provide strong phantom- and multi-site–based validation of the underlying GNL field estimation and correction strategies that we employ in the present work. However, the relevance and extent of these GNL correction effects in large, longitudinal brain aging cohorts, particularly those focused on mild cognitive impairment, remain less well characterized and motivate the present study.

In next section we first describe the clinical cohort and diffusion MRI acquisition, followed by the gradient nonlinearity correction and analysis steps used to address these six questions.

## 2. Methods

### 2.1. Study design

**Fig 1:**
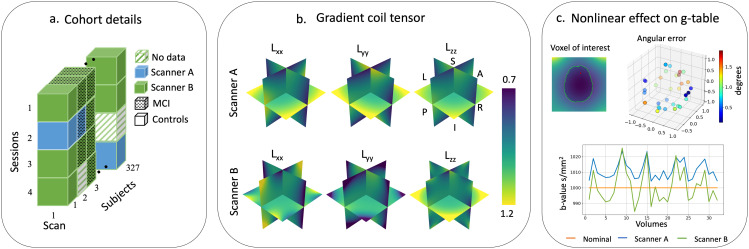
(a) The MCI clinical cohort comprises 327 participants, each undergoing up to four diffusion MRI sessions acquired on Scanner A (blue) or Scanner B (green). (b) Maps of the diagonal elements of the gradient nonlinearity tensor, L_xx_, L_yy_, and L_zz_, estimated from empirical phantom field maps for each scanner (FOV 384 × 384 × 384 mm). These entries describe the local scaling of nominal gradients applied along the x, y, and z axes and illustrate scanner-dependent spatial variation in effective diffusion weighting. (c) At a representative brain location (marked in panel b), the sphere plot shows the angular deviation between nominal and GNL-corrected b-vectors (example shown for Scanner B), and the line plot shows the corresponding effective b-values across diffusion volumes for Scanner A (blue) and Scanner B (green) relative to the nominal b-value (orange). Together, these panels summarize the cohort, the underlying gradient nonlinearity fields, and their impact on the effective diffusion encoding used in our analyses. Our study analyzes the effects of GNL on this clinical cohort.

This study provides a comprehensive evaluation of gradient nonlinearity (GNL) effects in brain diffusion tensor MRI using data from the Vanderbilt Memory & Aging Project (VMAP). The dataset comprises 327 older adults, each with up to four diffusion MRI sessions, for a total of 948 imaging sessions across multiple scanner configurations (Fig 1). We focus on a clinically well-characterized cohort that includes individuals with mild cognitive impairment (MCI) and cognitively unimpaired controls, allowing us to examine how GNL may bias the interpretation of aging-related changes and group differences in brain structure.

We employ a robust empirical field-mapping procedure [18] combined with state-of-the-art nonlinearity correction [19] technique to assess and correct GNL distortions (discussion in section 2.4). We then analyze both the uncorrected and GNL-corrected data at multiple levels:

- Microstructural metrics (FA, MD, and V1) at the voxel and regional level
- Intersession and interscanner effects, where available
- Subcortical and cortical regions of interest derived from structural segmentation
- Macrostructural tract metrics (volume, endpoint volume, surface area) derived from tractography

Within this framework, we address the following questions:

1. What is the extent of GNL-related changes in microstructural features across individual scans?
2. How do GNL effects vary between intersession scans acquired on different scanner types and configurations?
3. How do GNL effects differ across subcortical brain regions, and how might they influence comparisons between cognitively unimpaired and MCI participants?
4. How does GNL correction affect estimated associations between aging and diffusion metrics?
5. What are the GNL effects on macrostructural features in a tract-based analysis?
6. For typical GNL-related changes in FA and MD, how many participants would be required for these differences to become statistically detectable at the group level?

An overview of the cohort, scanner configurations, and gradient nonlinearity estimation is provided in Fig 1 and S1Table.

### 2.2. Clinical study cohort

The VMAP cohort [20] is a longitudinal study of older adults designed to investigate vascular health and brain aging. The study was approved by the Vanderbilt University Medical Center Institutional Review Board (IRB approval number 120158, valid from July 11, 2024, to July 10, 2025), and all participants provided written informed consent in accordance with institutional and ethical guidelines. All the IRB, ethics, and informed consent details are in the Supplementary document.

Participants were between 60 and 95 years of age and were required to speak English, have adequate auditory and visual acuity, and have a dependable study partner [20]. The cohort includes both cognitively unimpaired and MCI participants, with a balanced representation of males (n = 192) and females (n = 135), an average of 16 years of education, and approximately 86% non-Hispanic White [20]. Among cognitively unimpaired participants, cerebrospinal fluid (CSF) amyloid-β (Aβ42) concentrations average ~760 pg/mL, whereas in the MCI group levels average ~620 pg/mL; APOE ε4 carrier status is present in 41% of cognitively unimpaired participants and 30% of those with MCI [20]. This rich clinical and biomarker characterization has been used previously to relate white-matter microstructure to self-perceived cognitive deficits (20), making VMAP a suitable cohort for assessing the potential impact of GNL on diffusion-derived measures.

Each participant underwent 2–4 diffusion MRI sessions over longitudinal follow-up, resulting in a total of 948 diffusion scans. The mean follow-up time (i.e., time between imaging sessions) in the imaging sessions is 2.04 + /- 1.81 years. Individual sessions were acquired on one of several scanner configurations summarized in S1Table, with the diffusion MRI acquisition protocol held nominally constant across configurations as described in Section 2.3.

### 2.3. Diffusion MRI acquisition

Diffusion MRI data were acquired using a single-shell diffusion-weighted sequence with b = 1000 s/mm² and 32 diffusion-encoding directions, plus a single non–diffusion-weighted (b = 0 s/mm²) volume at the beginning of each scan. The field of view was 25.6 × 25.6 cm² with a 128 × 128 acquisition matrix, yielding an in-plane resolution of 2.0 × 2.0 mm². Sixty axial slices with 2.0 mm thickness provided whole-brain coverage. Typical timing parameters were a repetition time (TR) of approximately 10,000 ms, an echo time (TE) of approximately 60 ms, and a flip angle of 90°. Parallel imaging (SENSE) was used for all diffusion acquisitions, with an in-plane acceleration factor of 2. Scans were performed on Philips 3T Achieva systems using either an 8-channel SENSE head coil or a 32-channel dStream head coil. Scanner model, software version, head-coil configuration, and number of imaging session are listed for each configuration in Supplementary S1 Table.

### 2.4. Image processing steps

**Fig 2:**
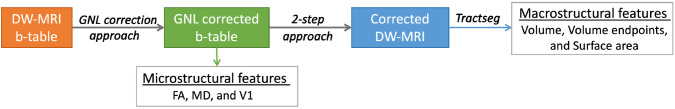
Outline of processing and measurement used in the study. We obtain the GNL corrected voxel-wise b-matrix using the SOTA (Bammer et al [19]) approach. Next, GNL corrected DW-MRI is generated through the two-step approximation method. We then examine the microstructural features and macrostructural features before and after GNL correction.

The raw diffusion MRI data is processed through denoising, intensity normalization, motion-induced artifact correction, susceptibility-correction, eddy current-correction, and slice-wise imputation using the PreQual pipeline [21] and quality assured. The processed data is corrected for gradient nonlinearity as detailed in the next section and shown in Fig 2. These preprocessing steps (including susceptibility- and eddy-current–distortion correction) address geometric distortions of the EPI images. The GNL correction described in Section 2.3 retrospectively adjusts the diffusion encoding (b-matrix and gradient directions) on the preprocessed images.

### 2.5. Nonlinearity gradient correction

Rogers et. al introduced an empirical field-mapping procedure using a large phantom, combined with solid harmonic approximations, to estimate the nonlinear gradient fields (denoted as L) [18]. These empirically estimated fields have been validated against vendor-provided theoretical gradient field data, demonstrating close alignment with the manufacturer's true fields [22]. Using the empirical filed-mapping approach, we estimated scanner-specific nonlinear gradient field maps for the scanners used in the VMAP cohort. The estimated nonlinear field are 3 x 3 L_ij_ describes the fractional deviation of the effective gradient along axis i due to a nominal gradient applied along axis j (i, j ∈ {x, y, z}), such that the actual gradient G_actual_ = (I + L) G_nominal_, at each spatial location.

Because GNL is a scanner hardware property that varies with position relative to magnet isocenter, we compute L field in scanner coordinate space using scanner-specific gradient fieldmaps. The resulting voxel-wise L was then transformed into each subject’s native diffusion space based on the subject’s head position and applied as a subject-specific correction.

Using the L field, the actual b-matrix is calculated with a state-of-the-art approach [19]. We then reconstruct GNL-corrected diffusion signals using the two-step signal approximation method, which (i) rescales the signal and (ii) resamples diffusion-encoding directions to account for gradient nonlinearities [23]. Throughout this manuscript, “GNL correction” refers specifically to this two-step correction using voxel-wise b-values and diffusion-encoding directions derived from the empirical gradient field maps; it does not introduce additional geometric dewarping beyond the standard distortion-correction steps described in Section 2.2.

The empirical field-mapping procedure used here has been extensively validated in prior work using large phantoms and solid harmonic approximations, demonstrating that the empirically estimated gradient fields closely match the manufacturer-provided fields and yield stable voxel-wise b-value and gradient direction estimates across repeated scans and scanners [22]. Multi-center phantom and cohort studies further show that applying empirical GNL correction reduces apparent diffusion coefficient bias and improves reproducibility across space, scanner platforms, and clinical protocols [13]. In addition, the two-step signal approximation technique used to reconstruct GNL-corrected DW-MRI signals has been validated against the full voxel-wise correction, showing negligible differences in microstructural model parameters and effect sizes, while enabling seamless integration into standard diffusion processing pipelines [23]. Thus, the combination of empirical field mapping and two-step signal approximation employed in this study builds directly on a body of phantom- and cohort-based validation work. Accordingly, our analysis focuses on characterizing the impact of these established corrections in a large aging and MCI cohort.

The VMAP cohort was scanned on two different scanners. Within a typical brain FOV of approximately 20 × 20 × 20 cm^3^ centered on isocenter, the diagonal elements of the gradient nonlinearity tensor were highly consistent between the two scanners. Mean percent differences were small for all components (L_xx_: 0.30%, L_yy_: 0.45%, L_zz_: − 0.07%), with 95th-percentile absolute differences of 2.36%, 3.15%, and 1.62%, respectively. Only isolated voxels showed larger discrepancies (maximum absolute percent differences up to 4.15% for L_xx,_ 10.6% for L_yy_, and 2.99% for L_zz_), while the underlying values for both scanners remained close to unity throughout the brain.

### 2.6. Microstructure and Macrostructure features

Microstructural and macrostructural features were computed for each diffusion MRI session. At the microstructural level, we fit a diffusion tensor model to the diffusion signal using linear least squares, yielding fractional anisotropy (FA), mean diffusivity (MD), and the primary eigenvector (V1) from both the GNL-uncorrected and GNL-corrected b-matrices. Although DTI is not the most biophysically detailed representation of tissue microstructure and cannot fully resolve complex fiber crossings or orientation dispersion, it remains the de facto standard for single-shell clinical protocols and large longitudinal cohorts, including VMAP [24]. Our goal in this work is therefore not to compare or optimize diffusion models, but to quantify how GNL affects the diffusion metrics that are actually available in typical clinical and retrospective datasets where only single-shell data exist. To partially address the angular sensitivity of GNL beyond the tensor model, we also evaluate changes in V1 and in tractography-derived macrostructural metrics, which depend on local fiber orientation and streamline geometry. While multi-shell, multi-compartment models can provide richer microstructural information and are theoretically preferable for detailed subcortical analyses, the reality is that most clinical and large cohort studies still rely on single-shell acquisitions, for which DTI remains the most feasible option [25–27].

Regional microstructural measures were derived using SLANT. [28]. SLANT (Spatially Localized Atlas Network Tiles) is a deep learning approach to segment the whole brain into 113 labels based on BrainCOLOR [29,30] protocol. The white matter (WM) and gray matter (GM) masks are derived from SLANT segmentations. FA values below 0.1 are biased due to noise [31], hence excluded in our analysis. Although we could concentrate on higher FA values, our study considered changes in both GM and WM. This is critical because our research centers on GNL implications in clinical research, where alterations in WM are significant [32–34].

For the regional analyses in Section 3.3, we computed mean FA, MD, and V1 within each of the 113 SLANT labels for every imaging session. We then summarized the GNL effect in each region by the median across sessions of the absolute difference between GNL-corrected and uncorrected metrics. In addition, our analysis focused on subcortical structures (e.g., diencephalon, basal ganglia, thalamus, and accumbens) and selected cortical regions implicated in aging and MCI.

Fiber orientation distribution functions (fODFs) were estimated from the diffusion data to characterize the local distribution of fiber orientations and to support tract-oriented density imaging (TODI). Whole-brain tractography was then performed on the fODFs for both the GNL-uncorrected and GNL-corrected datasets, and white-matter bundles were segmented using TractSeg, yielding 72 bundles in total [35]. From these, we retained 63 association, commissural, thalamic, striatal, and projection pathways that are particularly relevant to aging and cognition. Using the Sherbrooke Connectivity Imaging Lab (scilpy) toolbox [36], we computed bundle-wise macrostructural metrics, volume, endpoint volume, and surface area, for each bundle in each condition. Finally, we quantified the impact of GNL correction on tract geometry by calculating the angular correlation coefficient (ACC) and density correlation between the GNL-corrected and uncorrected tractograms for each bundle with scilpy. The tractography were performed in subject space, thereby decoupling hardware-position effects from anatomical location effects.

### 2.7. Statistical analysis

To quantify the magnitude of GNL-related differences between conditions (e.g., between scanner configurations and/or before vs. after GNL correction), we computed voxelwise standardized effect size maps using Cohen’s d [37], defined as


$$\begin{array}{c} d=(\mu_{\text{1}}-\mu_{\text{2}})/\sqrt{(\overset{\text{2}}{\underset{\text{1}}{\sigma }}+\overset{\text{2}}{\underset{\text{2}}{\sigma }})/\text{2}} \end{array}$$

where $\mu_{\text{1}},\mu_{\text{2}}$ and $\sigma_{\text{1}},\sigma_{\text{2}}$ denote the mean and standard deviation across subjects at each voxel for the two conditions being compared. The resulting Cohen’s d maps for interscanner comparisons summarize the standardized magnitude of GNL-related changes in FA and MD.

For longitudinal analyses of aging effects (Section 3.5), we fit region-wise linear mixed-effects (LME) [38] models with FA or MD as the dependent variable, age as the primary fixed-effect predictor, and a subject-specific random intercept to account for repeated imaging sessions per participant. For each region of interest, the model can be written as


$$\begin{array}{c} {\text{Metric}}_{ij}=\beta_{\text{0}}+\beta_{\text{1}}\;{\text{Age}}_{ij}+\beta_{\text{2}}\;{\text{Sex}}_{i}+u_{i}+\varepsilon_{ij}, \end{array}$$

where i indexes participants and j indexes imaging sessions, ${\text{Metric}}_{ij}$ is FA or MD, $\beta_{\text{0}}$ is the fixed intercept, and $\beta_{\text{1}}$ and $\beta_{\text{2}}$are the fixed-effect coefficients for age and sex, respectively. The term $u_{i}\sim N(\text{0},\overset{\text{2}}{\underset{u}{\sigma }})$ is a subject-specific random intercept and $\varepsilon_{ij}\sim N(\text{0},\sigma^{\text{2}})$ is the residual error. Models were fit separately for the GNL-uncorrected and GNL-corrected datasets using the MixedLM implementation in statsmodels [39]. For each ROI we extracted fixed-effect estimates, their standard errors, and corresponding z-values (estimate divided by standard error), obtained two-sided p-values from the standard normal distribution, and applied a Benjamini–Hochberg false discovery rate (FDR) correction to these fixed-effect p-values within each model. We focus on the z-values and associated (FDR-adjusted) p-values for the age term to assess whether GNL correction alters the estimated association between aging and diffusion metrics, considering p<0.05 (two-sided) as statistically significant.

### 2.8. Sample size estimation

To assess how large a study would need to be for the observed GNL-related changes in diffusion metrics to become statistically detectable at the group level, we performed a simple power analysis based on the within-subject differences between GNL-corrected and uncorrected data. For metric, we computed the subject-specific differences and summarized these by their sample mean $\mu_{\Delta }$ and standard deviation $\sigma_{\Delta }$. Assuming that these differences are approximately normally distributed, we then used power and sample size calculations [40] to calculate the number of participants required in a paired t-test to detect a non-zero mean difference $\mu_{\Delta }$ with a two-sided significance level α=0.05 and 80% power.

During the preparation of this work the authors used ChatGPT 3.5, an AI language model developed by OpenAI, in order to assist in rephrasing the text in this paper for clarification. After using this tool, the authors reviewed and edited the content as needed and takes full responsibility for the content of the publication.

## 3. Results

### 3.1. What is the extent of GNL in microstructure features across individuals?

**Fig 3:**
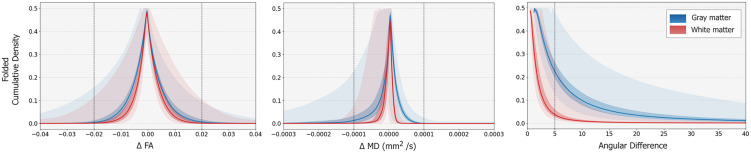
Distribution of GNL changes in microstructure features in the GM and WM across VMAP dataset show clear trends. The solid lines are median curve for GM and WM, and shaded bands indicate the interquartile range (darker shade) and full min–max range. GM shows consistently larger folded CDF values than WM for a given magnitude of change, indicating that a larger fraction of GM voxels is affected by GNL. The vertical dashed line indicates the threshold used in the text (e.g., 3.3% MD change), and the corresponding height of the curves can be interpreted as the fraction of voxels with at least that magnitude of GNL-related change. In FA, there are up to 20-30% of brain with 2% change. In MD, there is pronounced overestimation with up to 30% of brain with 3.3% change. In V1, up to 5 degrees changes in 50% of the brain.

Fig 3 summarizes the distribution of GNL-related changes in FA, MD, and V1 across the 948 imaging sessions in the VMAP dataset using folded cumulative distribution functions (fCDFs) for GM and WM. For each subject, we computed the folded cumulative distribution of voxelwise differences between GNL-corrected and uncorrected values and then summarized these across subjects by plotting the median curve for GM and WM, with shaded bands indicating the interquartile range and full min–max range. We observe the following: (1) The fraction of the brain volume where the FA difference is negative and greater in magnitude than 0.02 is between 0 and approximately 16% depending on the individual. While the FA difference is positive and greater in magnitude than 0.02 is between 0 and 10% of the brain volume depending on the individual. This suggests that about a 2% and more change in FA for 0–16% of the brain. (2) The portion of the brain volume exhibiting a negative MD difference greater than 0.1 µm²/ms spans from 0 to 30% among individuals, compared to 0–3% for a positive MD difference exceeding 0.1 µm²/ms. This suggest that a 3.3% and more change in MD for 5–20% of the brain. (3) The fraction of the brain volume where the angular difference exceeds 5 degrees ranges from 0 to approximately 50% depending on the individual, with gray matter consistently showing a higher affected fraction than white matter.

**Fig 4:**
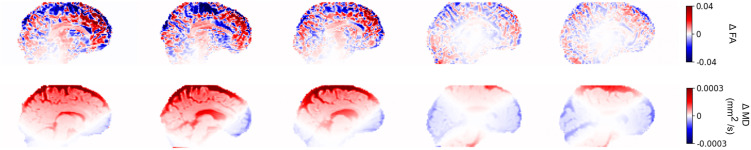
Each column corresponds to a different subject, highlighting the regional difference in response to GNL correction. Greater changes occur in regions further from the scanner's isocenter, influenced by the orientation and positioning of the brain.

Fig 4 illustrates difference map of changes in FA and MD before and after GNL. The first 3 subjects are who exhibit the most pronounced changes in the fCDF presented in Fig 3. Regions at the periphery of the brain demonstrate significant impacts. These effects are influenced by the orientation and positioning of the brain within the scanner, with areas further from the isocenter showing greater changes. We also note that FA is more sensitive than MD to modest orientation-dependent GNL changes, whereas MD tends to change more smoothly, which explains the alternating blue/red FA pattern across adjacent regions while MD appears more homogeneous.

### 3.2. How does the GNL effect vary between intersession scans with different scanner types?

**Fig 5:**
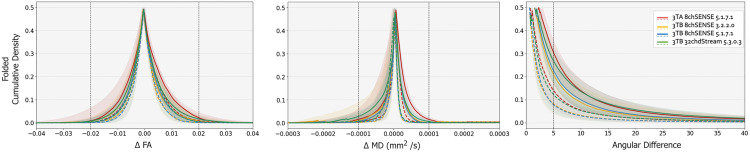
Folded cumulative distributions of GNL-related FA changes in GM and WM across intersession scanner configurations. For each scan pair with intersession observations, we computed the folded cumulative distribution of voxelwise differences (GNL-corrected minus uncorrected) separately for GM and WM, then summarized each scanner/coil configuration by the median curve with min–max shading. Solid lines denote GM; dashed lines denote WM. Scanner A (3TA 8chSENSE, red) shows slightly larger folded CDF values, indicating a larger fraction of voxels with a given magnitude of FA change than the three Scanner B configurations (yellow, blue, and green), consistent with stronger effective GNL effects in this subset. The vertical dashed line indicates the threshold used in the text (e.g., 0.02 FA), and the corresponding height of the curves can be interpreted as the fraction of voxels with at least that magnitude of GNL-related change.

Fig 5 extends the folded CDF analysis to intersession pairs acquired on different scanner and coil configurations (Scanner A: 3TA 8chSENSE; Scanner B: 3TB 8chSENSE with software versions 3.2.2.0 and 5.1.7.1; and 3TB 32ch dStream 5.3.0.3).We observe the following: (1) In 3TA, approximately 2% (0.02 dotted lines in Fig 5) or more change in FA affects up to 1% of the brain, whereas in 3TB, this change impacts about 0.5% of the brain. (2) Both 3TA and 3TB exhibit similar ranges when the MD difference is negative. The portion of the brain where the MD difference is positive and greater than 0.05 µm²/ms is 0–1% in 3TA and up to 0.5% in 3TB, suggesting about 0.5% more pronounced GNL effects in 3TA. (3) Both 3TA and 3TB exhibit similar effects for angular difference, with negligible difference between them, yet the GNL effect in each contribute to a change of 5 degrees or more in 30% of the brain. (4) Overall, the 3TB scanner with the 32chdStream coil demonstrates the least GNL effects, while the 3TA scanner using 8chSENSE coil shows the most in this intersession comparison. (5) The effect size Cohen's d for 3TA 8chSENSE and 3TB 32ch dStream are small before and after correction, indicating that the distribution is similar before and after GNL correction for interscanner observations (S1 Fig). The scan assignments were determined by clinical logistics (e.g., scanner availability and scheduling constraints). As a result, only about 2% of scans in this cohort were acquired on a different scanner. The interscanner results shown in this section are based solely on the 15 paired sessions from participants who were scanned on both Scanner A (3TA) and Scanner B (3TB), allowing a direct within-subject comparison between scanners despite the overall imbalance in the number of sessions per system.

### 3.3. How does the GNL effect differ in terms of subcortical brain regions in microstructure features and how does this contribute when comparing MCI and healthy controls?

Across all 113 regions, the median of the mean GNL-related changes in FA and MD is small, lying around 0.2% and 1.5%, respectively, whereas the median of the mean angular deviation reaches up to 7°, consistent with the angular differences observed in Figs 3 and 5. We therefore focus on regions at the extremes of this distribution, those with the highest positive and negative median GNL-related changes in FA, MD, and V1, as illustrative examples of contexts in which the GNL effect is comparable in magnitude to the disease-related differences between MCI and controls, rather than implying that only these specific structures are affected.

The mean changes in FA and MD are minimal, lying within the 0.5% and 1.5% respectively. In contrast mean change in angular difference reaches up to 15 degrees, supporting the angular difference observations made in Fig 3 and 5. The mean changes in FA, MD, and V1 in subcortical regions across 948 imaging sessions in the VMAP dataset, highlighting the medians with the highest positive and negative changes shown in S2 Fig.

The subregions exhibiting the top five highest positive and negative median changes in mean FA include left/right anterior orbital gyrus (AOrG), left gyrus rectus (GRe), left middle occipital gyrus (MOrG), left postcentral gyrus medial segment (MPoG), left/right inferior occipital gyrus (IOG), left occipital pole (OCP), right occipital fusiform gyrus (OFuG), and right cerebellum exterior. Of these IOG and OFuG are linked with visual memory – processing and recalling visual information, and recognizing face and complex objects, respectively. The median difference in FA between MCI and healthy controls in the right IOG is 0.001, where GNL shows a mean change of 0.0017. Similarly, the median FA difference in the right OFuG is 0.0005, with a mean change of 0.0017 in GNL effect, underlining its relevance in the comparison between MCI and healthy controls.

In terms of MD, the regions with the five highest positive and negative median change include the right anterior orbital gyrus (AOrG), right/left frontal pole (FRP), right/left lateral orbital gyrus (LOrG), left postcentral gyrus medial segment (MPoG), left precentral gyrus medial segment (MPrG), left superior frontal gyrus (SFG), and right/left supplementary motor cortex (SMC). The median difference in MD between MCI and healthy controls in the left FRP is 13.69 µm^2^/s, when the mean change in GNL effect of 42.616 µm^2^/s, in the right SFG the median difference is 20.83 µm^2^/s, with a mean change of 33.03 µm^2^/s in GNL effects, again emphasizing the significance of GNL.

The regions with the five highest positive median angular changes include the right/left middle frontal gyrus (MFG), right superior frontal gyrus (SFG), and right/left superior parietal lobule (SPL). We observe these superior regions, occipital, and parietal lobules have an effect of 7 degrees or more, aligning with the overall trends observed in angular changes across the dataset.

### 3.4. What is the effect of GNL in basal ganglia?

The GNL effect in FA within the basal ganglia, when comparing MCI to healthy controls, is quantified using masks derived from the diencephalon, putamen, caudate, pallidum, basal forebrain, thalamus, and accumbens regions, as defined by SLANT. The median absolute difference in FA between MCI and healthy controls in the basal ganglia is 0.0032, with the GNL effect accounting for 5% of this difference. While the median absolute difference in MD between MCI and healthy controls in the basal ganglia is 36.62 µm^2^/s, where GNL accounts for 0.33% of the effect. These findings suggest that while there are detectable microstructural changes in the basal ganglia associated with MCI, the influence of GNL on these changes is minimal.

### 3.5. What is the effect of correction for GNL in aging?

A linear mixed effects (LME) regression was conducted for each region before and after GNL correction for FA and MD to determine the association of aging on these microstructure measures. We do not observe any drastic change in z-value or statistical significance for before and after correction (S3 Fig).

### 3.6. What are the GNL effects in macrostructure features in a tract-based analysis?

**Fig 6:**
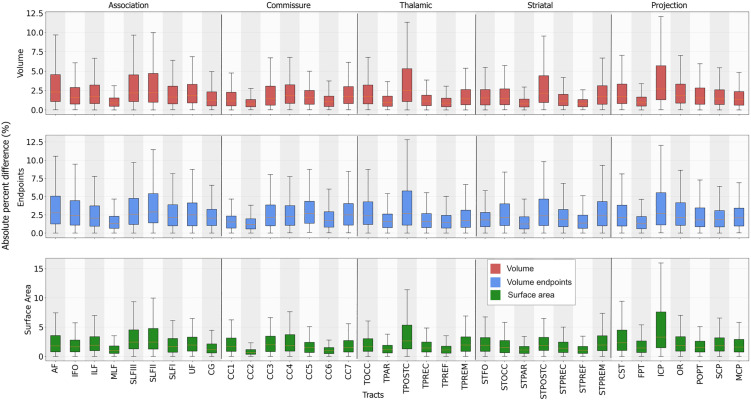
Absolute percent difference in tract-level macrostructural metrics before and after GNL correction. Boxplots show, for each white matter bundle, the distribution of absolute percent differences across sessions (left and right bundles combined) for volume (top), endpoints (middle), and surface area (bottom). Across bundles, the median absolute percent difference is approximately 3% for all three metrics, with some bundles showing comparatively larger changes in surface area and endpoints.

Fig 6 illustrates the GNL effects on the geometry of fiber bundles, focusing on volume, volume endpoints, and surface area. These metrics are essential for assessing the impact of GNL on fiber anatomy, since GNL induces spatial distortions that directly influence overall shape and size. The results highlight that the absolute percent difference for these metrics are below 12%, indicating that the GNL effects on the macrostructure features are relatively contained within this threshold. This observation suggests a modest influence of GNL on the structural properties of the fiber tracts. These percentage differences are comparable to, or smaller than, reported test–retest variability in tractography-based macrostructural measures [41]. Thus, for the single-shell 3T protocol studied here, the incremental impact of GNL on tract segmentation appears modest and is likely to be overshadowed by other sources of tractography variability in many applications.

**Fig 7:**
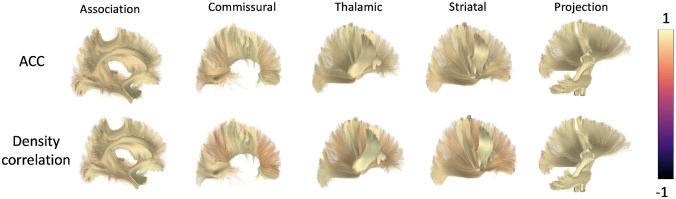
Mean ACC and density correlation for each bundle across participants show high correlation overall. However, the frontal regions exhibit comparatively lower correlation. The colors represent the correlation strength, ranging from negative to positive.

Fig 7 presents two distinct correlation measurements across brain regions using tract-oriented density imaging (TODI). The bundle-wise mean was calculated and averaged across participants for each bundle. Tractography and bundle segmentation were performed separately on the uncorrected and GNL-corrected diffusion data using identical reconstruction, tracking, and TractSeg parameters, so that any differences in bundle geometry reflect changes in the underlying diffusion signal rather than differences in processing settings. TractSeg is a convolutional neural network trained on multi-site diffusion data and has been shown to produce robust segmentations across scanners and acquisition protocols [35]. In the present context, this robustness implies that modest GNL-related intensity and orientation changes are more likely to perturb streamline geometry where they are spatially coherent than to induce unstable or spurious segmentations. This interpretation is consistent with the high angular correlation coefficients and density correlations observed in Fig 7. Finally, Angular correlation coefficient (ACC) maps compare the local orientation of bundles before and after GNL at each voxel. Most bundle regions show consistent fiber orientation. Patch-wise correlations between streamline density maps from both bundles show regions where densities coincide.

### 3.7. What sample size will make this difference significance?

Using this dataset, we first computed the within-subject differences between GNL-corrected and uncorrected metrics for each participant, $\Delta {\text{FA}}_{i\;}$ and $\Delta {\text{MD}}_{i}$, and treated these paired differences as approximately normally distributed. Tables 1 and 2 report the resulting number of participants (pairs of scans) required to reject the null hypothesis that the mean difference is zero under these assumptions. For example, a mean FA difference of 0.02 would be expected to reach statistical significance with 90% power in a study with 598 participants.

**Table 1:** Number of participants required for the FA deviation to be statistically significant along with the minimum and the maximum value of nonlinear field determinant that produces the FA deviation.

No. of participants	FA deviation	Minimum field determinant	Maximum field determinant
598	0.02	0.9629	1.0272
988	0.015	0.9518	1.0340
1936	0.01	0.9489	1.0417
5378	0.006	0.9452	1.0458
48395	0.002	0.9445	1.0486
48395	−0.002	0.9450	1.0494
5378	−0.006	0.9434	1.0469
1936	−0.01	0.9437	1.0461
988	−0.015	0.9474	1.0445
598	−0.02	0.9560	1.0256

**Table 2:** Number of participants required for the MD deviation to be statistically significant along with the minimum and maximum value of nonlinear field determinant that produces the MD deviation.

No. of participants	MD deviation (µm²/ms)	Minimum field determinant	Maximum field determinant
665	0.1	1.0399	1.0455
1099	0.075	1.0401	1.0411
2154	0.05	1.0244	1.0474
5981	0.025	1.0083	1.0444
5981	−0.025	0.9437	0.9924
2154	−0.05	0.9507	0.9814
1099	−0.075	0.9533	0.9731
665	−0.1	0.9437	1.0410

To relate these effect sizes back to the physical magnitude of gradient nonlinearity, we also examined the determinant of the nonlinear field. For each target FA or MD deviation, we identified voxels across all participants whose GNL-related change in FA or MD matched that deviation and recorded the minimum and maximum values of determinant in those voxels. The ranges of determinant associated with each effect size are shown in the last two columns of Tables 1 and 2.

These power calculations are intentionally conservative and isolate the contribution of GNL-related changes under otherwise idealized assumptions. In real-world studies, additional sources of variability such as residual head motion, scanner drift, segmentation bias, and physiological noise will also contribute to measurement variance. The sample sizes reported in Tables 1 and 2 should therefore be interpreted as indicating when GNL effects become statistically detectable if all other factors are controlled, not as fixed thresholds at which GNL correction becomes universally required.

In this dataset, the variations in head position for translations along the x, y, and z axes range from significant motions of approximately 10 mm-20 mm from the reference mean b0 of a random participant, while others exhibit smaller shifts of about 0 mm-10 mm. Similarly, rotations along the x, y, and z axes show deviations ranging from 5–10° in some brains compared to the reference, whereas most others range from 0–5 degrees, as depicted in S4 Fig. These patterns are consistently observed in intersession scans (3TB 8chSENSE 3.2.2.0 and 3TB 8chSENSE 5.1.7.1; 3TB 8chSENSE 3.2.2.0 and 3TA 8chSENSE 5.1.7.1) as well (S4 Fig).

## 4. Discussion

This study demonstrates the impact of gradient nonlinearity (GNL) correction on both microstructural (FA, MD, V1) and macrostructural diffusion MRI measures in a clinical aging and MCI cohort. Across brain regions and major white-matter bundles, we observed systematic GNL-related changes in FA, MD, V1 and in tractography-derived measures (e.g., tract volume, surface area, apparent connectivity correlation (ACC), and density correlation). While some differences in FA, MD, and V1 were also observed between scanner configurations, the overall pattern of results indicates that the dominant driver of GNL effects in this dataset is subject positioning relative to isocenter, rather than receive-coil differences or software updates within the same site.

### 4.1. Sources of variability

The findings in this paper should be interpreted in the broader context of diffusion MRI reproducibility. Inter- and intra-site variability arises from various sources such as scanner hardware (including but not limited to imaging gradient nonlinearity, the magnetic field homogeneity, shimming, and sensitivity of the coil), software versions (pulse sequences and reconstruction algorithms), and operating environments (number of head coil channels, head positions) across different sites [42,43]. Variability in FA across different sites can reach about 5% in WM and 25% in GM [44], whereas in conditions like schizophrenia, variations typically hover around 5% [42]. Some other hardware related sources in DW-MRI are signal drift (17% variation in signal intensity across a session) and Gibbs ringing (9% variation in signal intensity) [5]. Shim errors, imaging inconsistencies, and eddy currents introduce minor ADC errors around 5%, whereas GNL effects can range from −55% (SI) and 25% (RL) when measured offset from the isocenter [13].

Within this landscape, GNL is distinctive because it introduces a spatially structured, position-dependent bias that increases with offset from isocenter and is, in principle, correctable via voxel-wise diffusion-encoding correction. Prior work also indicates that GNL correction does not interact adversely with noise at typical clinical SNR levels, supporting the view that GNL effects can be isolated and addressed without significant interference from noise [45]. Thus, the ability to isolate and effectively correct for GNL effects in the presence of noise enhances the accuracy of diffusion MRI metrics, affirming the potential of these GNL correction.

In our dataset, the main hardware differences between configurations (S1 Table) are head-coil type (8-channel SENSE vs 32-channel dStream) and scanner software version; the underlying imaging gradients and GNL fields are determined by the gradient system and are therefore independent of the receive coil. The coil primarily modulates SNR and the effectiveness of parallel imaging rather than the spatial pattern of GNL itself. As a result, the GNL estimation and correction pipeline is identical across configurations, and differences in SNR are interpreted as a separate source of variance that adds to, but does not confound, the GNL effects quantified here.

### 4.2. Consistency with prior work and interpretation of spatial patterns

Previous studies such as Bammer et al. have indicated that typical effect sizes in absolute diffusivity can vary from underestimations of approximately 10–15% near the center and to overestimations greater than 20% at the periphery within a standard clinical field-of-view of 25 cm for a 1.5T GE scanner [19]. For a 3T Siemens Skyra Connectome scanner, Mesri et al. observed significant angular discrepancies, ranging between 0 and 5°, and gradient amplitude with deviations up to 10% [9]. Moreover, the study highlighted accumulation of tract error as fiber trajectories propagate and that GNL can alter the significance of the test results across various brain regions [9]. For a 300 mT/m gradient Connectome scanner brain scan positioned 3 cm offset from the isocenter, mean diffusivity is underestimated by as much as 30% [10]. This scanner also exhibited angular deviation in fiber orientation, initially exceeding 3° at the edges [8]. Taken together, these studies show that GNL effects scale with distance from isocenter and can be amplified on ultra-strong gradient systems and in analyses that depend on orientation and tract propagation

Consistent with this literature, our 3T single-shell data show measurable but generally modest GNL-related effects. In Fig 3, with head positions varying approximately 0–10 mm and 0–10°, the observed effect sizes reach up to ~10% in MD and ~4% in FA, and FA variability attributable to GNL reaches ~2% over ~10–20% of the brain. We find ~10% changes in mean diffusivity which support previous findings [9]. Within this single-site cohort, we do not observe strong evidence that differences in head coils, scanners, or software updates produce effects comparable to the GNL-related shifts. GNL effect observed for the small subset of 3TA sessions likely reflects a combination of gradient nonlinearity and relatively larger offsets from isocenter in those specific scans, whereas many 3TB scans were acquired closer to isocenter.

Overall, these distributions indicate that, for this single-shell 3T protocol, GNL correction produces voxelwise changes in FA and MD that are generally small in absolute magnitude and comparable to or smaller than reported inter-session variability for diffusion metrics, particularly in white matter [41,44,45]. As we discuss in Section 4.3-4.4, the practical importance of such changes depends on the scientific question, the regions of interest, and the scale of the study.

The FA maps show adjacent regions of overestimation and underestimation (red and blue), whereas MD tends to exhibit more spatially uniform shifts within a given subject. This is consistent with prior tensor-simulation work [46], which demonstrated that nonlinear gradients can produce both increases and decreases in FA depending on the underlying tensor orientation and anisotropy, while MD changes are largely governed by the local determinant of nonlinear field and thus are more monotonic dependent on FA. In other words, small orientation differences between neighboring voxels can flip the sign of the FA bias, whereas the corresponding MD bias remains of the same sign but varies gradually in magnitude.

### 4.3. Implication for regional analyses and tractography

The frontal and occipital regions we highlight (e.g., SFG, FRP, IOG) were chosen because they fall among the regions with the largest median GNL-related changes when ranking all 113 SLANT labels, and because of their established roles in cognitive, working memory, decision making, and attention, and visual processing. In these areas, the MCI–control FA differences are very small (≈0.0005–0.001), and the MD contrasts are modest (on the order of 10–20 µm²/s), whereas the corresponding GNL-related shifts in FA and MD are of similar or greater magnitude (roughly 0.001–0.002 for FA and 30–40 µm²/s for MD). These regions should therefore be viewed as illustrative “worst-case” examples, in which uncorrected GNL is of similar magnitude to, and in these specific cases slightly larger than, the subtle disease-related effects in functionally important frontal and occipital areas.

In the basal ganglia, we find nonlinear correction changes are about 5% in FA and 0.33% in MD of that of MCI and healthy controls. These regions are particularly critical for Alzheimer’s disease (AD) research due to their involvement in cognitive functions, their susceptibility to vascular changes, and their potential role in early disease detection and progression [47–49]. Together, these observations suggest that, while GNL can modulate estimates in anatomically important deep gray structures, its relative contribution to the observed MCI–control differences in this cohort is modest.

The effect sizes introduced by GNL across the population in our study do not undermine the validity of previous studies without GNL correction. As shown in S2 Fig, the median of average changes in FA and MD across sub-cortical regions are minimal, at 0.2% and 1.5% respectively, while the median of mean angular deviation is substantive up to 7°. Aging studies include association, commissure, thalamic, striatal, and projection tracts to assess cognitive functions, sensory processing, and motor skills. These tracts help understand changes due to aging and neurodegenerative diseases, highlighting declines in interhemispheric communication, sensory and motor control, and cognitive processing [50,51]. In this context, our macrostructural analysis of tract volume, endpoint volume, and surface area provides a direct way to assess whether GNL materially alters the interpretation of tract-level changes in aging and MCI. However, Figs 6 and 7 indicate that the impacts on tractography are minor and are likely overshadowed by tractography’s inherent reproducibility errors. This obscures the changes with GNL correction and unlikely affect clinical tractography. Furthermore, there were no significant correlations between log determinant of nonlinear field and absolute percent difference in bundle measures.

### 4.4. Practical considerations

As researchers continue to advance the sensitivity of diffusion MRI techniques through higher gradient amplitudes, increased slew rates, reduced echo spacing, the ability to retrofit advanced coils into existing systems, refined analysis methods, and larger sample sizes, it becomes increasingly important at least to quantify and, when feasible, correct for the effects of GNL [41,52]. Our simple power calculations (Tables 1 and 2) illustrate that even the modest GNL-related shifts in FA and MD observed here would become statistically detectable in cohorts of several hundred to a few thousand participants. However, statistical significance in such large samples does not by itself establish clinical importance; the decision to prioritize GNL correction should be made in light of the expected effect sizes, other sources of measurement variability, and the specific scientific or clinical question.

A practical consideration for Philips users is that the vendor’s Diffusion Excellence Pack includes the Low Variance (LOVA) ADC [53] option, which automatically corrects apparent diffusion coefficient (ADC) maps for gradient linearity errors and improves the reproducibility of ADC measurements over a large field of view. These corrections are applied in-line to scalar ADC maps, but they do not propagate to diffusion tensor–derived quantities such as FA, tensor eigenvectors, or tractography outputs, which are typically computed offline from the raw diffusion-weighted images. Consequently, even on systems where vendor-supplied ADC/GNL correction is available, retrospective b-matrix–based GNL correction, as implemented here, remains necessary when interpreting FA, V1, and tractography-based metrics.

### 4.5. Limitations

This study has several limitations. First, we examined gradient nonlinearity effects for two 3T scanner systems (98% of scans on a single system) from a single institution, both using similar single-shell diffusion protocols (b = 1000 s/mm², 32 directions). By design, our data therefore primarily sample variability in head positioning and orientation relative to the same underlying gradient coil, rather than differences between fundamentally different gradient designs. The GNL effects quantified here should thus be interpreted mainly as position-dependent biases on one gradient system (plus minor protocol and coil differences). The magnitude and spatial pattern of GNL, and therefore the impact of GNL correction, may differ for other vendors, field strengths, or multi-shell/high-b acquisitions [9].

Second, while our GNL correction pipeline is based on previously validated empirical field maps and b-matrix correction methods, we did not perform additional phantom experiments specifically for this clinical dataset, and residual errors in the estimated gradient fields may persist. We also acknowledge a limitation of the empirical GNL mapping procedure used in this study. The large voxelwise discrepancies near the FOV edges between the two empirical GNL tensor maps (up to 10.6% for individual L_yy_ voxels) are likely driven by non-GNL measurement errors, such as chronic shim gradients, rather than true hardware differences between the two nominally identical Achieva systems. Although the empirical mapping approach was validated against vendor-provided theoretical GNL fields by Hansen et al., [22] non-GNL contamination sources such as chronic shim gradients were not explicitly screened for in the VMAP scanner fieldmaps. Because 98% of scans were acquired on a single scanner and mean inter-scanner differences across the brain FOV were small (L_xx_: 0.30%, L_yy_: 0.45%, L_zz_: −0.07%), this is unlikely to affect the present results. Future multi-system studies should nonetheless explicitly characterize and remove such non-GNL contamination prior to empirical field estimation.

Third, our analyses are based on DTI-level metrics (FA, MD, V1) derived from a single-shell acquisition, which matches the clinical protocol of the VMAP cohort. While advanced multi-shell and multi-compartment models can provide richer microstructural information and may in principle exhibit different sensitivities to GNL, such models are not available for this dataset and are not yet standard in routine clinical practice. Importantly, recent work using higher-order models and spherical deconvolution has reported GNL-related biases of similar order in orientation and tractography metrics, particularly for data acquired far from isocenter and on ultra-strong gradient systems [8–10]. In this context, the DTI-based effects we report should be viewed both as a characterization of GNL in real-world single-shell clinical data and as a conservative lower bound on the potential influence of GNL in more sophisticated diffusion models. Fourth, the cohort consists of older adults enrolled in a memory and aging study; the generalizability of these findings to younger populations or other clinical indications (e.g., acute stroke, traumatic brain injury) remains to be established. Finally, we did not explicitly quantify the test–retest reliability of our tractography pipeline (TractSeg) under GNL perturbations; therefore, some portion of the observed macrostructural differences may reflect segmentation variability rather than purely geometric effects of GNL.

### 4.6. Future work

Future work could extend this framework to other diffusion models and connectome-level analyses to further characterize the impact of GNL correction A natural extension of this work would be to use the empirically observed distributions of head translations and rotations in VMAP (S4 Fig), together with the measured gradient nonlinearity tensors field, to perform forward simulations of GNL bias under different scanner-usage and multi-system scenarios. For a single gradient system, one could place a standardized brain anatomy at each observed head position, apply the corresponding nonlinear tensor field, and compute predicted voxelwise changes in b-values, MD, and diffusion-encoding directions, yielding bias histograms for MD and orientation in the anatomies of interest. The same framework can be extended to explore more realistic multi-scanner and multi-system settings. For example, one could generate balanced scenarios in which each subject is scanned equally often on both VMAP systems (1:1 or 2:2 designs) or construct synthetic multi-center studies by pairing the VMAP head-position distributions with published gradient fields from additional systems such as Connectome-like ultra-strong gradient coils. Implementing and validating these multi-system simulations is beyond the scope of the present clinical analysis, but they represent a natural next step for generalizing our findings to broader multi-center diffusion MRI settings.

In summary, while the overall effect sizes observed were modest, the accuracy gained through GNL correction can influence clinical interpretations and scientific conclusions, particularly in large-scale, multi-site studies where even subtle inaccuracies can cumulatively alter outcomes. Future work should continue to refine GNL correction methods and explore their implications in larger cohorts to bolster the reliability and accuracy of studies.

## Supporting information

S1 Fig The intersession effect is small with and without correction. (DOCX)

S2 Fig Region specific variation in mean FA, MD, and V1 after GNL correction. Median with highest positive and negative change are highlighted in green and red respectively. Although these values are statistically significant, the difference in FA and MD become significant when comparing MCI with healthy controls in highlighted ROIs. Mean angular difference in superior regions are as high as 20 degrees. The underlying numerical data are provided in the attached CSV file as S2 Table.
(DOCX)

S3 Fig The absolute *z-value* for each independent LME regression grouped by region-type. Blocks marked with an asterisk represent associations meeting the pFDR < 0.05 threshold. No drastic change in z-value after GNL correction for 948 imaging sessions.
(DOCX)

S4 Fig Head position variability across VMAP imaging sessions, showing translations (mm) and rotations (degrees) along the x (red), y (green), and z (blue) axes relative to a common reference b0 volume. Each data point represents one imaging session. Translations range from approximately 0–10 mm and rotations from approximately 0–10°, with a subset of sessions showing larger shifts of up to 10–20 mm. Both intrasession and intersession distributions are shown, confirming that head positioning variability is consistent across scanner configurations.
(DOCX)

S1 Table MRI scanner specifications, software versions, and the number of imaging sessions per configuration in the study. (DOCX)

S2 Table Region specific variation in mean FA, MD, and V1 after GNL correction. (CSV)

## References

[1] Le Bihan D, Iima M. Diffusion magnetic resonance imaging: What water tells Us about biological tissues. PLoS Biol. 2015;13(7):e1002203. doi: 10.1371/journal.pbio.1002203 26204162 PMC4512706

[2] Benzinger TLS, Jindal S. MR Imaging of neurodegeneration. Molecular Imaging of Neurodegenerative Disorders. Springer International Publishing. 2023. 169–81. doi: 10.1007/978-3-031-35098-6_11

[3] Kantarci K, Avula R, Senjem ML, Samikoglu AR, Zhang B, Weigand SD, et al. Dementia with Lewy bodies and Alzheimer disease: neurodegenerative patterns characterized by DTI. Neurology. 2010;74(22):1814–21. doi: 10.1212/WNL.0b013e3181e0f7cf 20513818 PMC2882217

[4] Zhang Y, Schuff N, Du A-T, Rosen HJ, Kramer JH, Gorno-Tempini ML, et al. White matter damage in frontotemporal dementia and Alzheimer’s disease measured by diffusion MRI. Brain. 2009;132(Pt 9):2579–92. doi: 10.1093/brain/awp071 19439421 PMC2732263

[5] Tax CMW, Bastiani M, Veraart J, Garyfallidis E, Okan Irfanoglu M. What’s new and what’s next in diffusion MRI preprocessing. Neuroimage. 2022;249:118830. doi: 10.1016/j.neuroimage.2021.118830 34965454 PMC9379864

[6] Budrys T, Veikutis V, Lukosevicius S, Gleizniene R, Monastyreckiene E, Kulakiene I. Artifacts in magnetic resonance imaging: how it can really affect diagnostic image quality and confuse clinical diagnosis?. J vibroeng. 2018;20(2):1202–13. doi: 10.21595/jve.2018.19756

[7] Hurd RE, Deese A, Johnson MO, Sukumar S, van Zijl PCM. Impact of differential linearity in gradient-enhanced NMR. Journal of Magnetic Resonance, Series A. 1996;119(2):285–8. doi: 10.1006/jmra.1996.0089

[8] Guo F, de Luca A, Parker G, Jones DK, Viergever MA, Leemans A, et al. The effect of gradient nonlinearities on fiber orientation estimates from spherical deconvolution of diffusion magnetic resonance imaging data. Hum Brain Mapp. 2021;42(2):367–83. doi: 10.1002/hbm.25228 33035372 PMC7776002

[9] Mesri HY, David S, Viergever MA, Leemans A. The adverse effect of gradient nonlinearities on diffusion MRI: From voxels to group studies. Neuroimage. 2020;205:116127. doi: 10.1016/j.neuroimage.2019.116127 31476431

[10] Rudrapatna U, Parker GD, Roberts J, Jones DK. A comparative study of gradient nonlinearity correction strategies for processing diffusion data obtained with ultra-strong gradient MRI scanners. Magn Reson Med. 2021;85(2):1104–13. doi: 10.1002/mrm.28464 33009875 PMC8103165

[11] Tan ET, Marinelli L, Slavens ZW, King KF, Hardy CJ. Improved correction for gradient nonlinearity effects in diffusion-weighted imaging. J Magn Reson Imaging. 2013;38(2):448–53. doi: 10.1002/jmri.23942 23172675

[12] Malyarenko DI, Ross BD, Chenevert TL. Analysis and correction of gradient nonlinearity bias in apparent diffusion coefficient measurements. Magn Reson Med. 2014;71(3):1312–23. doi: 10.1002/mrm.24773 23794533 PMC3823647

[13] Malyarenko DI, Newitt DC, Amouzandeh G, Wilmes LJ, Tan ET, Marinelli L, et al. Retrospective correction of ADC for gradient nonlinearity errors in multicenter breast DWI trials: ACRIN6698 multiplatform feasibility study. Tomography. 2020;6(2):86–92. doi: 10.18383/j.tom.2019.00025 32548284 PMC7289257

[14] Newitt DC, Tan ET, Wilmes LJ, Chenevert TL, Kornak J, Marinelli L, et al. Gradient nonlinearity correction to improve apparent diffusion coefficient accuracy and standardization in the american college of radiology imaging network 6698 breast cancer trial. J Magn Reson Imaging. 2015;42(4):908–19. doi: 10.1002/jmri.24883 25758543 PMC4629811

[15] Tao AT, Shu Y, Tan ET, Trzasko JD, Tao S, Reid RD, et al. Improving apparent diffusion coefficient accuracy on a compact 3T MRI scanner using gradient nonlinearity correction. J Magn Reson Imaging. 2018;48(6):1498–507. doi: 10.1002/jmri.26201 30255963 PMC6263730

[16] Mulkern RV, Ricci KI, Vajapeyam S, Chenevert TL, Malyarenko DI, Kocak M, et al. Pediatric brain tumor consortium multisite assessment of apparent diffusion coefficient z-axis variation assessed with an ice-water phantom. Acad Radiol. 2015;22(3):363–9. doi: 10.1016/j.acra.2014.10.006 25435183 PMC4323850

[17] Rogers BP, Blaber J, Welch EB, Ding Z, Anderson AW, Landman BA. Stability of gradient field corrections for quantitative diffusion MRI. Proc SPIE Int Soc Opt Eng. 2017;10132:101324X. doi: 10.1117/12.2254609 28736467 PMC5521266

[18] Rogers BP, Blaber J, Newton AT, Hansen CB, Welch EB, Anderson AW, et al. Phantom-based field maps for gradient nonlinearity correction in diffusion imaging. Proc SPIE Int Soc Opt Eng. 2018;10573:105733N. doi: 10.1117/12.2293786 29887658 PMC5990280

[19] Bammer R, Markl M, Barnett A, Acar B, Alley MT, Pelc NJ, et al. Analysis and generalized correction of the effect of spatial gradient field distortions in diffusion-weighted imaging. Magn Reson Med. 2003;50(3):560–9. doi: 10.1002/mrm.10545 12939764

[20] Jefferson AL, Gifford KA, Acosta LMY, Bell SP, Donahue MJ, Davis LT, et al. The vanderbilt memory & aging project: Study design and baseline cohort overview. J Alzheimers Dis. 2016;52(2):539–59. doi: 10.3233/JAD-150914 26967211 PMC4866875

[21] Cai LY, Yang Q, Hansen CB, Nath V, Ramadass K, Johnson GW, et al. PreQual: An automated pipeline for integrated preprocessing and quality assurance of diffusion weighted MRI images. Magn Reson Med. 2021;86(1):456–70. doi: 10.1002/mrm.28678 33533094 PMC8387107

[22] Hansen CB, Rogers BP, Schilling KG, Nath V, Blaber JA, Irfanoglu O, et al. Empirical field mapping for gradient nonlinearity correction of multi-site diffusion weighted MRI. Magn Reson Imaging. 2021;76:69–78. doi: 10.1016/j.mri.2020.11.005 33221421 PMC7770121

[23] Kanakaraj P, Cai LY, Yao T, Rheault F, Rogers BP, Anderson A, et al. Efficient approximate signal reconstruction for correction of gradient nonlinearities in diffusion-weighted imaging. Magn Reson Imaging. 2023;102:20–5. doi: 10.1016/j.mri.2023.03.014 36965836 PMC10517071

[24] Sanvito F, Kaufmann TJ, Cloughesy TF, Wen PY, Ellingson BM. Standardized brain tumor imaging protocols for clinical trials: Current recommendations and tips for integration. Front Radiol. 2023;3:1267615. doi: 10.3389/fradi.2023.1267615 38152383 PMC10751345

[25] Martín-Martín C, Planchuelo-Gómez Á, Guerrero ÁL, García-Azorín D, Tristán-Vega A, de Luis-García R, et al. Viability of AMURA biomarkers from single-shell diffusion MRI in clinical studies. Front Neurosci. 2023;17:1106350. doi: 10.3389/fnins.2023.1106350 37234256 PMC10208402

[26] Yao T, Archer DB, Kanakaraj P. Learning-based free-water correction using single-shell diffusion MRI. In: SPIE, 2024. 36–42. 10.1117/12.3006901 PMC11394251 39281711

[27] Teller N, Chad JA, Wong A. Single-shell diffusion MRI for imaging white-matter microstructure in COVID-19: DTI vs. correlated diffusion imaging. Journal Title. 2023.

[28] Huo Y, Xu Z, Xiong Y, Aboud K, Parvathaneni P, Bao S, et al. 3D whole brain segmentation using spatially localized atlas network tiles. Neuroimage. 2019;194:105–19. doi: 10.1016/j.neuroimage.2019.03.041 30910724 PMC6536356

[29] Klein A, Tourville J. 101 labeled brain images and a consistent human cortical labeling protocol. Front Neurosci. 2012;6:171. doi: 10.3389/fnins.2012.00171 23227001 PMC3514540

[30] Klein A, Dal Canton T, Ghosh SS, Landman B, Lee J, Worth A. Open labels: Online feedback for a public resource of manually labeled brain images. 2010.

[31] Seo Y, Wang ZJ, Morriss MC, Rollins NK. Minimum SNR and acquisition for bias-free estimation of fractional anisotropy in diffusion tensor imaging - A comparison of two analytical techniques and field strengths. Magn Reson Imaging. 2012;30(8):1123–33. doi: 10.1016/j.mri.2012.04.015 22819179

[32] de Groot M, Cremers LGM, Ikram MA, Hofman A, Krestin GP, van der Lugt A, et al. White matter degeneration with aging: Longitudinal diffusion MR imaging analysis. Radiology. 2016;279(2):532–41. doi: 10.1148/radiol.2015150103 26536311

[33] Madden DJ, Bennett IJ, Song AW. Cerebral white matter integrity and cognitive aging: Contributions from diffusion tensor imaging. Neuropsychol Rev. 2009;19(4):415–35. doi: 10.1007/s11065-009-9113-2 19705281 PMC2787975

[34] Guttmann CR, Jolesz FA, Kikinis R, Killiany RJ, Moss MB, Sandor T, et al. White matter changes with normal aging. Neurology. 1998;50(4):972–8. doi: 10.1212/wnl.50.4.972 9566381

[35] Wasserthal J, Neher P, Maier-Hein KH. TractSeg - Fast and accurate white matter tract segmentation. Neuroimage. 2018;183:239–53. doi: 10.1016/j.neuroimage.2018.07.070 30086412

[36] Renauld E, Boré A, Poirier C, Valcourt-Caron A, Karan P, Théberge A, et al. Tractography analysis with the scilpy toolbox. Aperture Neuro. 2026. doi: 10.52294/001c.154022

[37] Cohen J. Statistical power analysis for the behavioral sciences. Routledge. 2013.

[38] Laird NM, Ware JH. Random-effects models for longitudinal data. Biometrics. 1982;38(4):963–74. doi: 10.2307/2529876 7168798

[39] Seabold S, Perktold J. Statsmodels: Econometric and statistical modeling with python. SciPy. 2010;7(1):92–6.

[40] Dupont WD, Plummer WDJ. Power and sample size calculations: A review and computer program. Controlled Clinical Trials. 1990;11(2):116–28. 2161310 10.1016/0197-2456(90)90005-m

[41] Gudino N, Littin S. Advancements in gradient system performance for clinical and research MRI. J Magn Reson Imaging. 2023;57(1):57–70. doi: 10.1002/jmri.28421 36073722

[42] Mirzaalian H, Ning L, Savadjiev P, Pasternak O, Bouix S, Michailovich O, et al. Inter-site and inter-scanner diffusion MRI data harmonization. Neuroimage. 2016;135:311–23. doi: 10.1016/j.neuroimage.2016.04.041 27138209 PMC5367052

[43] Jovicich J, Marizzoni M, Bosch B, Bartrés-Faz D, Arnold J, Benninghoff J, et al. Multisite longitudinal reliability of tract-based spatial statistics in diffusion tensor imaging of healthy elderly subjects. Neuroimage. 2014;101:390–403. doi: 10.1016/j.neuroimage.2014.06.075 25026156

[44] Vollmar C, O’Muircheartaigh J, Barker GJ, Symms MR, Thompson P, Kumari V, et al. Identical, but not the same: Intra-site and inter-site reproducibility of fractional anisotropy measures on two 3.0T scanners. Neuroimage. 2010;51(4):1384–94. doi: 10.1016/j.neuroimage.2010.03.046 20338248 PMC3163823

[45] Kanakaraj P, Cai LY, Rheault F, Yehe F-C, Rogers BP, Schilling KG, et al. Mapping the impact of nonlinear gradient fields with noise on diffusion MRI. Magn Reson Imaging. 2023;98:124–31. doi: 10.1016/j.mri.2023.01.004 36632947 PMC10275501

[46] Kanakaraj P, Hansen CB, Rheault F. Mapping the impact of non-linear gradient fields on diffusion MRI tensor estimation. SPIE, 2022. 1203203. 10.1117/12.2611900 PMC9604130 36303581

[47] Bown CW, Khan OA, Liu D, Remedios SW, Pechman KR, Terry JG, et al. Enlarged perivascular space burden associations with arterial stiffness and cognition. Neurobiol Aging. 2023;124:85–97. doi: 10.1016/j.neurobiolaging.2022.10.014 36446680 PMC9957942

[48] Gogniat MA, Khan OA, Bown CW, Liu D, Pechman KR, Taylor Davis L, et al. Perivascular space burden interacts with APOE-ε4 status on cognition in older adults. Neurobiol Aging. 2024;136:1–8. doi: 10.1016/j.neurobiolaging.2024.01.002 38280312 PMC11384903

[49] Robb WH, Kim DS, Houston ML, Pechman KR, Verde AR, Davis LT, et al. Arterial spin‐labeling spatial coefficient of variation is associated with cerebral small vessel disease. Alzheimer’s & Dementia. 2023;19(S17). doi: 10.1002/alz.080534

[50] Archer DB, Schilling K, Shashikumar N, Jasodanand V, Moore EE, Pechman KR, et al. Leveraging longitudinal diffusion MRI data to quantify differences in white matter microstructural decline in normal and abnormal aging. Alzheimers Dement (Amst). 2023;15(4):e12468. doi: 10.1002/dad2.12468 37780863 PMC10540270

[51] Schilling KG, Archer D, Yeh F-C, Rheault F, Cai LY, Hansen C, et al. Aging and white matter microstructure and macrostructure: A longitudinal multi-site diffusion MRI study of 1218 participants. Brain Struct Funct. 2022;227(6):2111–25. doi: 10.1007/s00429-022-02503-z 35604444 PMC9648053

[52] Foo TKF, Tan ET, Vermilyea ME, Hua Y, Fiveland EW, Piel JE, et al. Highly efficient head-only magnetic field insert gradient coil for achieving simultaneous high gradient amplitude and slew rate at 3.0T (MAGNUS) for brain microstructure imaging. Magn Reson Med. 2020;83(6):2356–69. doi: 10.1002/mrm.28087 31763726

[53] Li Q, Zhao X, Wu P. Low Variance ADC Technique Reduces Bias of Kidney ADC Values. presented at: Proceedings of the 2022 ISMRM & ISMRT Annual Meeting and Exhibition; London, United Kingdom; 2022.

