# Supplementary figures and images for: Is correction for gradient nonlinearity necessary in a brain diffusion tensor MRI clinical study?
Source: PLoS One. 2026 Jul 6;21(7):e0350808. doi: 10.1371/journal.pone.0350808 (PMC13336164; doi:10.1371/journal.pone.0350808)

**
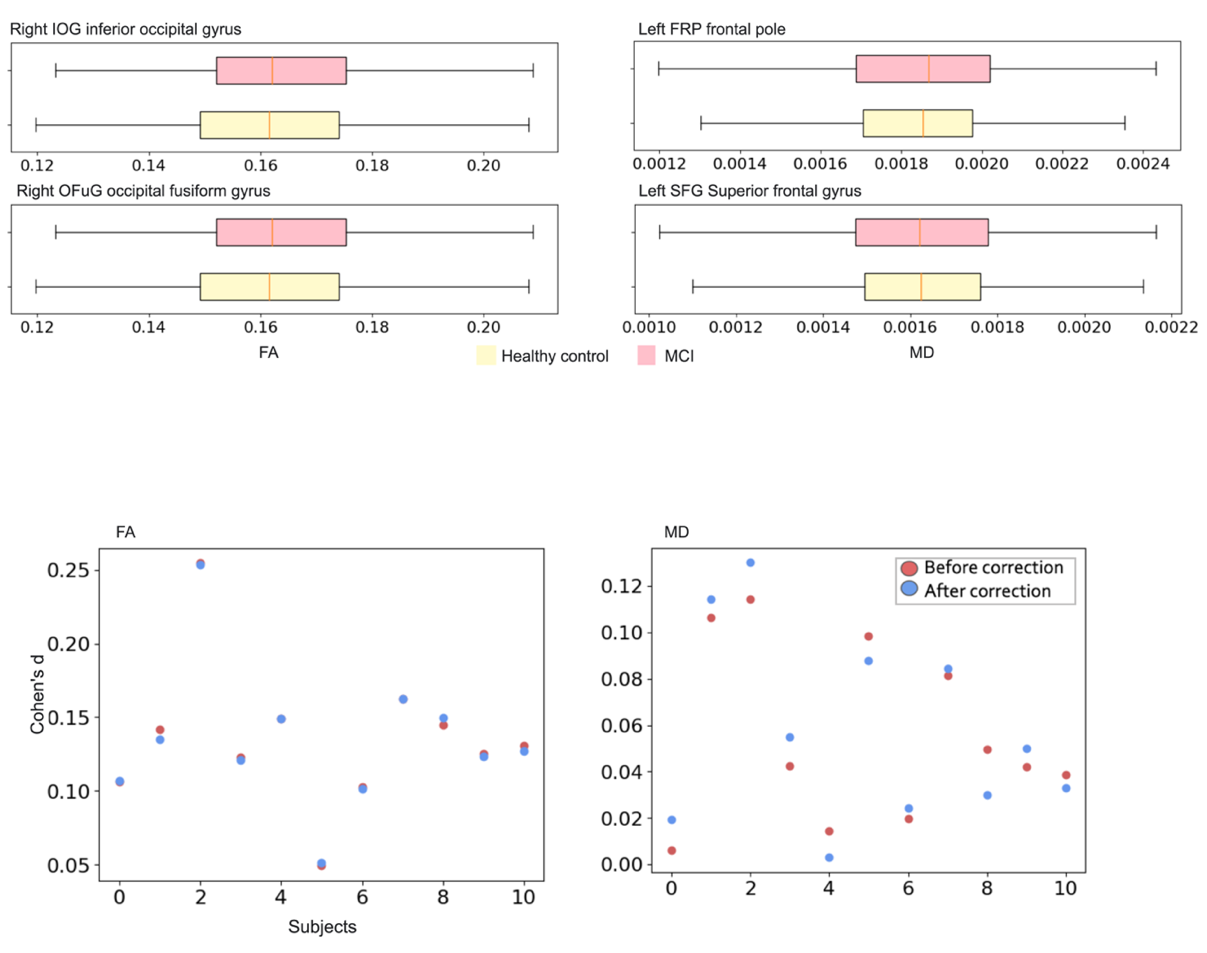
**

**Figure S1. The intersession effect is small with and without correction.**

Supplement: S1 Fig — (DOCX) [file pone.0350808.s001.docx]
