# Supplementary material for: Is correction for gradient nonlinearity necessary in a brain diffusion tensor MRI clinical study?
Source: PLoS One. 2026 Jul 6;21(7):e0350808. doi: 10.1371/journal.pone.0350808 (PMC13336164; doi:10.1371/journal.pone.0350808)
Supplement: S2 Fig — Median with highest positive and negative change are highlighted in green and red respectively. Although these values are statistically significant, the difference in FA and MD become significant when comparing MCI with healthy controls in highlighted ROIs. Mean angular difference in superior regions are as high as 20 degrees. The underlying numerical data are provided in the attached CSV file as S2 Table. (DOCX) [file pone.0350808.s002.docx]

**
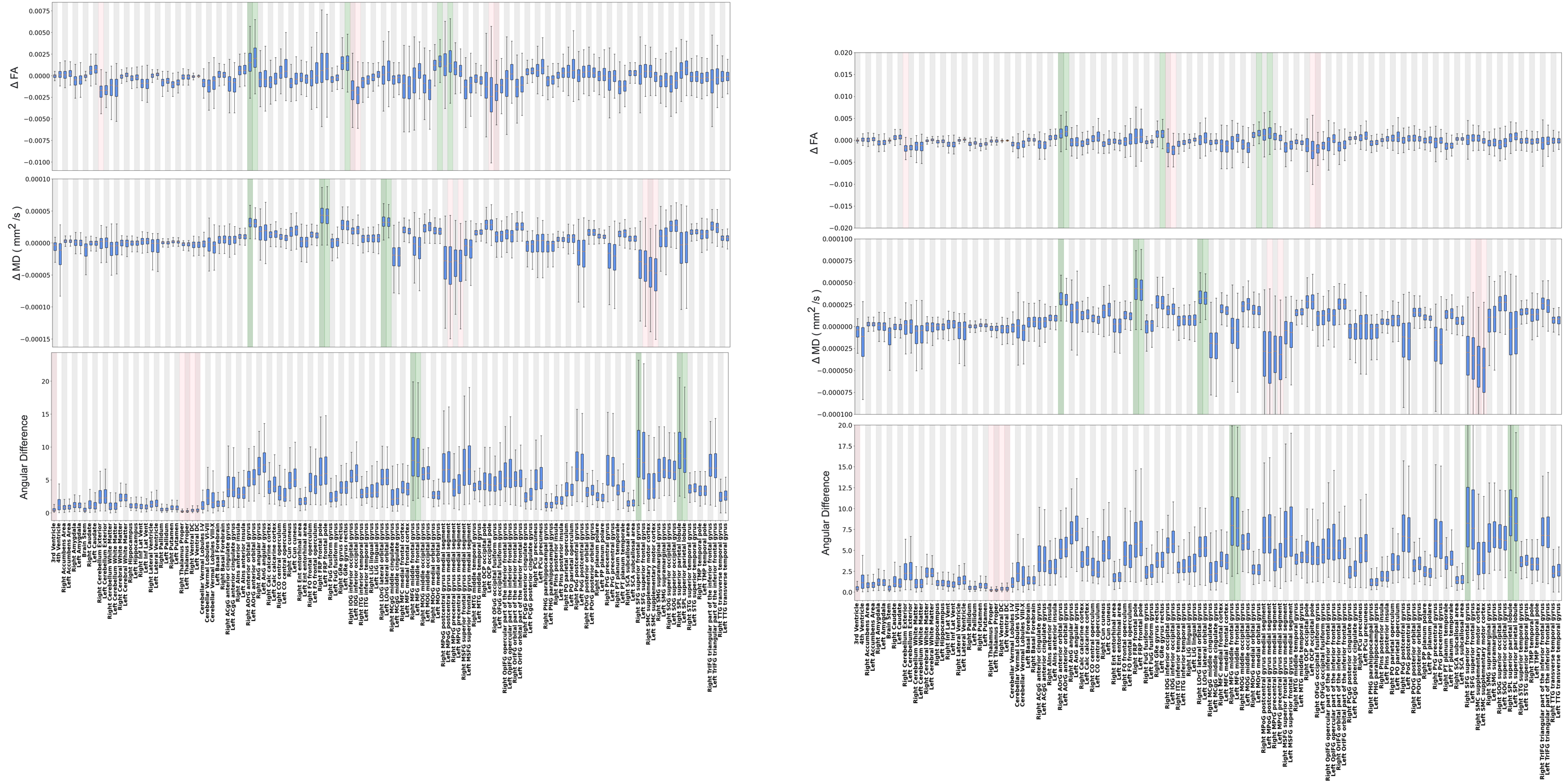
**

**Figure S2.** **Region specific variation in mean FA, MD, and V1 after GNL correction. Median with highest positive and negative change are highlighted in green and red respectively. Although these values are statistically significant, the difference in FA and MD become significant when comparing MCI with healthy controls in highlighted ROIs. Mean angular difference in superior regions are as high as 20 degrees. The underlying numerical data are provided in the attached CSV file as Table S2**.
