# Supplementary material for: Is correction for gradient nonlinearity necessary in a brain diffusion tensor MRI clinical study?
Source: PLoS One. 2026 Jul 6;21(7):e0350808. doi: 10.1371/journal.pone.0350808 (PMC13336164; doi:10.1371/journal.pone.0350808)
Supplement: S3 Fig — Blocks marked with an asterisk represent associations meeting the pFDR < 0.05 threshold. No drastic change in z-value after GNL correction for 948 imaging sessions. (DOCX) [file pone.0350808.s003.docx]

**
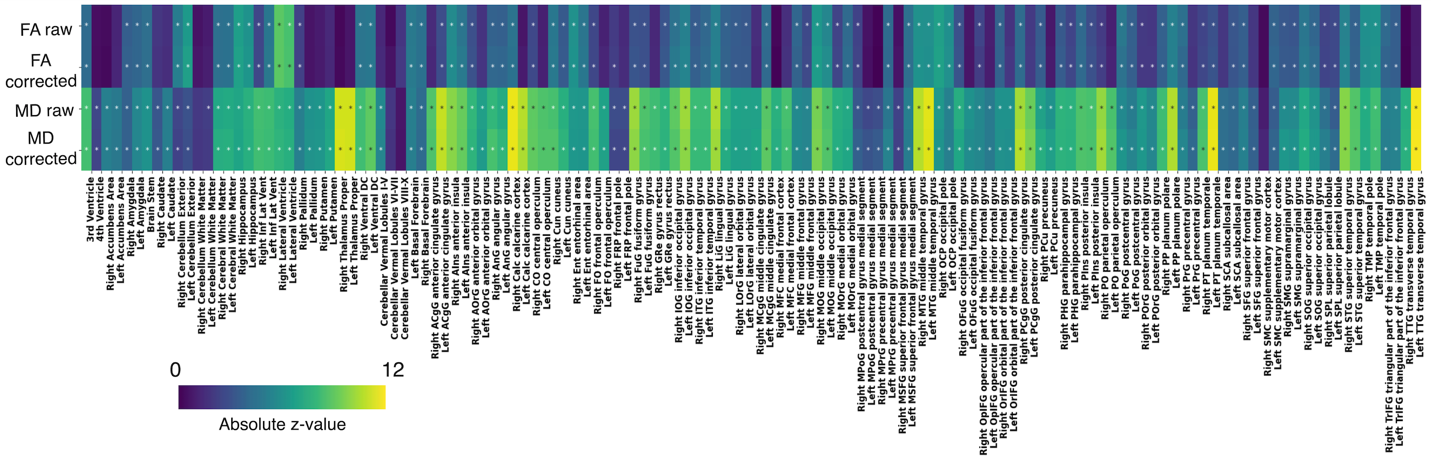
**

**S3. The absolute *z-value* for each independent LME regression grouped by region-type. Blocks marked with an asterisk represent associations meeting the pFDR < 0.05 threshold.** **No drastic change in z-value after GNL correction for 948 imaging sessions.**
