# Supplementary material for: Is correction for gradient nonlinearity necessary in a brain diffusion tensor MRI clinical study?
Source: PLoS One. 2026 Jul 6;21(7):e0350808. doi: 10.1371/journal.pone.0350808 (PMC13336164; doi:10.1371/journal.pone.0350808)
Supplement: S4 Fig — Translations range from approximately 0–10 mm and rotations from approximately 0–10°, with a subset of sessions showing larger shifts of up to 10–20 mm. Both intrasession and intersession distributions are shown, confirming that head positioning variability is consistent across scanner configurations. (DOCX) [file pone.0350808.s004.docx]

**
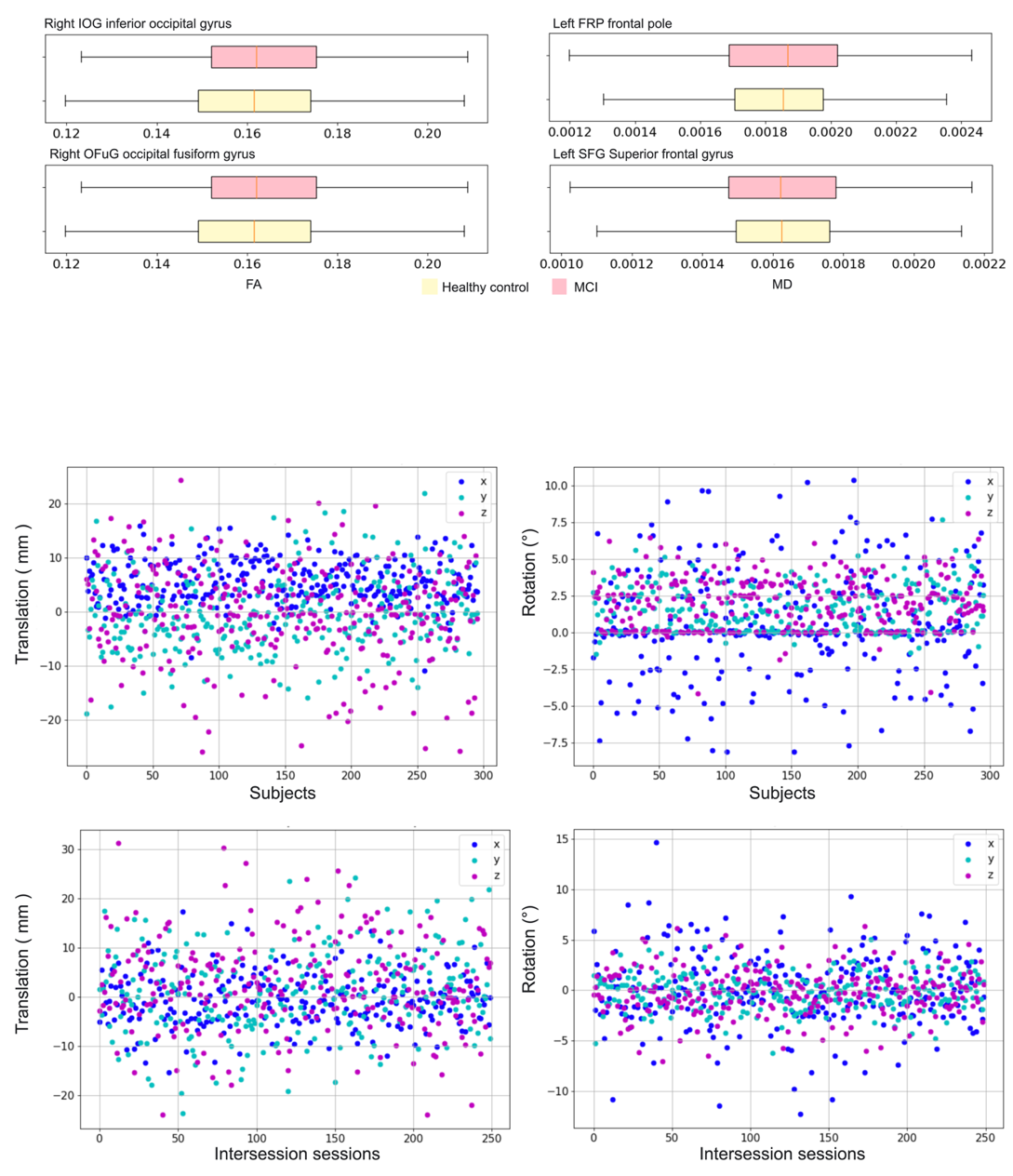
**

**Figure S4. Head position variability across VMAP imaging sessions, showing translations (mm) and rotations (degrees) along the x (red), y (green), and z (blue) axes relative to a common reference b0 volume. Each data point represents one imaging session. Translations range from approximately 0–10 mm and rotations from approximately 0–10°, with a subset of sessions showing larger shifts of up to 10–20 mm. Both intrasession and intersession distributions are shown, confirming that head positioning variability is consistent across scanner configurations.**
