# Supplementary material for: Is correction for gradient nonlinearity necessary in a brain diffusion tensor MRI clinical study?
Source: PLoS One. 2026 Jul 6;21(7):e0350808. doi: 10.1371/journal.pone.0350808 (PMC13336164; doi:10.1371/journal.pone.0350808)
Supplement: S1 Table — (DOCX) [file pone.0350808.s005.docx]

**Table S1: MRI scanner specifications, software versions, and the number of imaging sessions per configuration in the study.**

| Scanner ID | Scanner | Magnetic strength | Coil type | Software version | Imaging sessions |
| --- | --- | --- | --- | --- | --- |
| A | Philips Acheiva | 3T | 8chSENSE | 5.1.7.1 | 15 |
| B | Philips Acheiva | 3T | 8chSENSE | 3.2.2.0 | 351 |
| B | Philips Acheiva | 3T | 8chSENSE | 5.1.7.1 | 240 |
| B | Philips Acheiva | 3T | 32chdStream | 5.3.0.3 | 300 |
| B | Philips Acheiva | 3T | 32chdStream | 5.6.1.0 | 7 |
| B | Philips Acheiva | 3T | 8chSENSE | 5.3.0.2 | 24 |
| B | Philips Acheiva | 3T | 8chSENSE | 5.3.0.1 | 11 |
